# Supplementary material for: Metabolic and transcriptional activities underlie stationary-phase Pseudomonas aeruginosa sensitivity to Levofloxacin
Source: Microbiol Spectr. 2023 Dec 11;12(1):e03567-23. doi: 10.1128/spectrum.03567-23 (PMC10896071; doi:10.1128/spectrum.03567-23)
Supplement: Supplemental material — Supplemental methods, Tables S1 to S4, Figures S1 to S11, and captions for Videos S1 to S9. [file spectrum.03567-23-s0001.docx]

**SUPPLEMENTAL MATERIALS**

**Metabolic and Transcriptional Activities Underlie Stationary-Phase *Pseudomonas aeruginosa* Sensitivity to Levofloxacin**

Patricia J. Hare, Juliet R. Gonzalez, Ryan M. Quelle, Yi I. Wu, Wendy W.K. Mok

Supplemental Methods

Tables

Table S1

Table S2

Table S3

Table S4

Figures

Figure S1

Figure S2

Figure S3

Figure S4

Figure S5

Figure S6

Figure S7

Figure S8

Figure S9

Figure S10

Figure S11

Supplemental Video Captions

Video S1

Video S2

Video S3

Video S4

Video S5

Video S6

Video S7

Video S8

Video S9

References

**SUPPLEMENTAL METHODS**

**Culture Media and Antibiotics**

Cells were inoculated from -80 ^o^C frozen stocks (in 25% glycerol) into 16 mm diameter test tubes with 2 mL nutrient-rich media: cation-adjusted Mueller-Hinton Broth (CA-MHB) for *P. aeruginosa* or lysogeny broth (LB) for *E. coli*. CA-MHB was prepared from BD Difco Mueller Hinton Broth powder and cation-adjusted to final concentrations of 10 mg/L Mg^2+^ and 20 mg/L Ca^2+^; cations were prepared as 85 g/L MgCl_2_•6H_2_O and 28 g/L CaCl_2_ stock solutions in water and filter-sterilized before adding to autoclaved MHB media. LB was prepared with 10 g/L Bacto tryptone, 5 g/L Bacto yeast extract, and 10 g/L NaCl and autoclaved before use.

In order to decrease possible confounding factors due to batch variation of rich media, cultures for survival assays were grown in chemically defined minimal media. Basal Salt Media (BSM) with succinate as the sole carbon source was used for *P. aeruginosa* assays (1, 2). BSM was prepared in water with 30.8 mM K_2_HPO_4_, 19.3 mM KH_2_PO_4_, 15 mM (NH_4_)_2_SO_4_, 1 mM MgCl_2_, 2 μM FeSO_4_, and 15 mM succinic acid, then filter-sterilized before use. Gutnick media with 10 mM glucose was used for *E. coli* assays (3, 4). Gutnick-glucose media was prepared to final concentrations of 34.56 mM KH_2_PO_4_, 77.59 mM K_2_HPO_4_, 5.74 mM K_2_SO_4_, 0.406 mM MgSO_4_•7H_2_O, 10 mM NH_4_Cl, and 10 mM glucose as the sole carbon source, then filter-sterilized before use.

Antibiotics prepared in water were filter-sterilized using 0.22 μm polyethersulfone (PES) filters before use. Levofloxacin (Levo) was prepared as a 5 mg/mL stock solution in MilliQ water and titrated with 1 M NaOH until soluble. Tobramycin (TOB) was prepared as a 20 mg/mL stock solution in MilliQ water. Aztreonam (AZT) was prepared as a 40 mg/mL stock solution in DMSO. Thiolutin (Enzo Life Sciences) was prepared as a 1 mg/mL stock solution in DMSO. 2,2'-bipyridine was prepared as a 0.3 M stock in 200-proof ethanol. Thiourea was prepared as a 1.5 M stock in water and was sterilized by filtration through a syringe-driven filter with 0.22 μm pores. [5,6-^3^H]-uridine was purchased from PerkinElmer/Revvity (Hopkinton, MA, USA).

**Cloning of *recA* Deletion Strains**

Knockout strains of *P. aeruginosa* were generated using allelic exchange (5). The 500 base pairs upstream and downstream of *recA* in PAO1 or PA14 were cloned into a single gene fragment by overlap PCR using primers (see **Table S3**) that contained restriction sites for digestion and ligation into the *sacB* suicide vector, pEX18Gm. *P. aeruginosa* was transformed via conjugation by triparental mating with *E. coli* XL1-Blue containing the modified pEX18Gm-recAKO and *E. coli* HB101::pRK2013. After triparental mating, transformed *P. aeruginosa* were selected for on LB agar containing Irgasan (25 μg/mL) and Gentamicin (Gent; 75 μg/mL). Colonies on LB-Irgasan-Gent plates were patched onto LB-Gent agar or LB agar with Gent and 10% sucrose. Gent-resistant, sucrose-sensitive clones were cured of pEX18Gm by growing in liquid LB at 37 ^o^C for 6 h. After curing, clones were diluted 100-10,000-fold and plated to both LB agar and LB agar with 10% sucrose. Sucrose-resistant colonies were patched onto LB-10% sucrose agar and LB-Gent-10% sucrose agar. Gent-sensitive, sucrose-resistant clones were grown in LB media and screened for successful *recA* deletion by colony PCR using internal and external primer pairs as listed in **Table S3**.

**Whole Genome Sequencing (WGS) of *recA* Deletion Strains**

One clone each of PAO1 Δ*recA* and PA14 Δ*recA* was sent for whole genome sequencing at the UConn Center for Genome Innovation (CGI). Genomic DNA was extracted from each strain using the Qiagen Blood & Tissue Kit, according to the manufacturer’s protocols. Libraries were prepared by the CGI with the Illumina Nextera XT Library Prep Kit and sequenced on the Illumina Novaseq 6000 (150 bp paired end reads, 8 million reads per sample). Reads were trimmed using fastx_trimmer, aligned to the respective reference genomes using bowtie2, and finally indexed and formatted into bam files using samtools. Aligned reads were visualized in Integrative Genomics Viewer (IGV) browser sessions with the respective reference genomes. WGS reads are deposited in the NCBI Sequence Read Archive under BioProject number PRJNA1034140.

**Minimum Inhibitory Concentration Assays**

Antibiotic minimum inhibitory concentrations (MICs) were determined by the MIC test strip method (**Fig. S1**). *P. aeruginosa* strains were inoculated from -80 ^o^C frozen stocks (in 25% glycerol) into 2 mL test tubes of CA-MHB and grown at 37 ^o^C, shaking at 250 rpm, for 16 h. Following overnight growth, cells were pelleted by centrifugation (21,000 x g, 3 min), washed once in sterile 0.85% NaCl then resuspended to an OD_600_ of 0.2-0.3 in 0.85% NaCl. The resuspended cells were spread onto circular CA-MHB agar plates using sterile cotton swabs; cells were spread throughout the agar plate three times, rotating the agar plate 60 degrees each time. After the plates were dry, antibiotic MIC test strips (Liofilchem) were laid onto the surface at the geometric center of the plate and plates were inverted and incubated overnight (16-20 h) at 37 ^o^C. MICs were interpreted as the lowest drug concentration at which the lawn of bacterial growth did not intersect the test strip. Liofilchem antibiotic test strips were obtained through ThermoFisher: Aztreonam (22778040), Tobramycin (22777909), Levofloxacin (22777855).

The MICs of thiolutin, thiourea, and 2,2'-bipyridine were determined by broth microdilution method following CLSI standards. Cells were grown overnight in CA-MHB then diluted in test tubes with 2 mL CA-MHB to an OD_600_ 0.01-0.04 for growth to exponential phase. At OD_600_ 0.2-0.4, cells were diluted to 10^5^ cells/mL in CA-MHB and 100 μL was added to each well of a 96-well plate already containing 100 μL 2x-concentrated drug in CA-MHB. Drugs were serially diluted two-fold for testing the following final concentration ranges: 0.25 - 256 μg/mL thiolutin (*P. aeruginosa*), 0.03 - 32 μg/mL thiolutin (*E. coli*), 0.59 - 600 mM thiourea, and 0.04 - 40 mM 2,2'-bipyridine.

**Time-Lapse Microscopy Experiments**

Cells were prepared for imaging during Levo treatment by inoculating from frozen stocks into rich media (CA-MHB for *P. aeruginosa* or LB for *E. coli*). After 4-5 h pre-growth, inocula were diluted into 250 mL baffled flasks containing 25 mL of chemically defined media (BSM for *P. aeruginosa* or Gutnick-glucose for *E. coli*) and a 2 mL test tube with 2.5 μM SytoxBlue dye (**Fig. S1**). After 16 h of growth, cell-free conditioned media (CFCM) was collected from the flask by pelleting 10 mL of cells by centrifugation at 21,000 x g for 3 min. Then, the supernatant was filtered through a sterile PES filter (0.22 μm pore size). The agarose pads on which cells were seeded for imaging were made from 2 mL CFCM with 1.5% (w/v) molecular-grade agarose (Bio-Rad 1613101), 16 μM propidium iodide (Component B from the LIVE/DEAD BacLight Bacterial Viability and Counting Kit, Invitrogen L34856), and 5 μg/mL Levo. The agarose was poured into a Bioptechs Interchangeable Coverglass Dish with a sealed 30 mm round coverslip (#1.5 glass) in the bottom. It was then loosely covered with a 25 mm round coverslip to stabilize the agarose pad while allowing air exchange. After the pads solidified, the cells pre-grown in chemically defined media with SytoxBlue were diluted 30-fold in PBS for seeding onto the agarose pads and, once dried into the pad, a new 30 mm coverslip was placed against the cells. The sample was kept at 37 ^o^C during imaging in a PeCon live cell incubation chamber with a humidifying lid to prevent the pad from dehydrating and shifting.

Samples were imaged using a customized Zeiss Axiovert 200M microscope with a Plan-Apochromat 63x/1.40 Oil Ph3 M27 objective. MetaMorph Premier version 7.10.5 (Molecular Devices) was used to control the motorized components of the microscope and image acquisition. The phase contrast channel was used to search and maintain the focal plane during multi-dimensional acquisition using MetaMorph’s built-in autofocus algorithm. A Lumencor Spectra 7 LED light engine was used to excite samples for fluorescence imaging of propidium iodide (555/28 nm) and SytoxBlue (440/20 nm); Zeiss filter set 15 (Beamsplitter FT580, Emission LP590) and set 51 (Beamsplitter 470, Emission BP485/30) were used to image propidium iodide and SytoxBlue, respectively. Images were acquired on a pco.panda 4.2 bi sCMOS camera (6.5 μm pixel size) or Hamamatsu ORCA-R2 camera (6.45 μm pixel size). Images were taken every 10 min for 24 h.

**Time-Lapse Image Analysis**

Images were analyzed using Fiji (ImageJ2 version 2.9.0/1.53t). Phase channel image stacks were shade-corrected using BaSiC, merged with the OFP channel (for propidium iodide fluorescence), then the merged stacks were drift-corrected using the Correct_3D_Drift.py script (6, 7). Complete videos of the 24 h imaging are included in the supplemental file. For quantitative analysis, stacks were simplified by creating substacks with frames corresponding to t=0, 1, 3, 5, 7, and 24 h.

Cell tracking and morphological/fluorescent signal classification were conducted with the MicrobeJ plugin (version 5.13l) (8). The scale was set to 0.1032 μm/pixel. In brief, *P. aeruginosa* bacterial cells were detected on the Phase channel with the following parameters: Area (0.25-15 um), Length (0.1-20 um), Width (0-1 um), Curvature (0-0.7), Angularity (0-0.3), Exclude on Edges, Shape Descriptors, and Segmentation. The Tracking option with Lineage analysis was selected. For detection of propidium iodide positive cells (PI+), the Maxima tab was set to detect on the OFP channel in Foci mode with the following parameters: Tolerance (980), Z-score (15), Exclude on Edges, Shape Descriptors, Segmentation, Overlapping, and Inside (0.2 um Tolerance).

From the Results table, 100 numbers were randomly selected from the total number of bacteria in the first frame (t=0) using a random number generator. Those 100 cells were tracked through frames corresponding to t=1, 3, 5, 7, and 24 h and marked as PI+/- and intact or lysed (lysis was determined visually as a dramatic loss of rod-shaped morphology and cell volume and/or loss of phase contrast).

**Phage Plaque Assays**

To collect cell lysates for the phage plaque assay, *P. aeruginosa* PAO1 and PA14 were inoculated, cultured overnight, and treated with Levo as detailed above for the persistence assays. Before antibiotic treatment and after 7 h of treatment with Levo or water (for the treatment-free control), supernatant was collected from each culture and filtered through a sterile syringe-driven filter with 0.22 μm pores. These filtrates were stored at 4 ^o^C before they were used in the plaque assays.

For the plaque assays, 50 μL of overnight PAO1 and PA14 cultures were combined with 10 mL of top agar (0.8% LB-agar with 10 mM MgSO_4_) that has been cooled to ~50 ^o^C. The top agar was overlaid on 1.5% LB agar. The filtrates were serially diluted in SM buffer (100 mM NaCl, 8 mM MgSO_4_, 50 mM Tris HCl, pH 7.5, and 0.01% gelatin). As positive controls, bacteriophages JBD90, which preferentially infects PAO1, and JBD23, which preferentially infects PA14, were also serially diluted in SM buffer (9). 10 μL of each filtrate/bacteriophage dilution was spotted onto the bacteria-containing top agar. The plates were incubated at 37 ^o^C for 16 h before plaque forming units (PFUs) were enumerated.

**Measuring ATP Concentration with BactiterGlo**

*P. aeruginosa* was inoculated and cultured to exponential phase or stationary phase (16, 24, or 48 h) as described above. At designated times, the OD_600_ of each culture was measured and 100 μL of each sample was adjusted to an OD_600_ of 0.1 in succinate-free BSM and aliquoted to a well of an opaque, white LUMITRAC 96-well plate (Grenier Bio-One). 100 μL BactiterGlo (Promega) was added to each well and incubated in the dark at room temperature for 5 min before measuring luminescence on a Biotek multimode plate reader. For each replicate, a six-point standard curve from 0 to 1 μM ATP was prepared to calculate the concentration of ATP in each sample.

**Measuring Succinate Concentration via Fluorometric Assay**

*P. aeruginosa* PAO1 and PA14 were cultured in 25 mL of BSM to exponential phase (OD_600_ ~0.1-0.4) or stationary phase (16, 24, or 48 h) as detailed earlier. After measuring the OD_600_ of each culture, 500 μL culture was sampled for cell-free conditioned media (CFCM) collection: cells were pelleted by centrifugation at 21,000 x g and the supernatant was sterilized by passing it through a syringe-driven filter with 0.22 μm pores. CFCM samples were stored at -20 ^o^C until time for analysis.

The succinic acid assay reaction mixture was prepared following the manufacturer’s instructions (Sigma MAK335). In a black, clear-bottom 96-well plate, 20 μL of each CFCM was mixed with 80 μL of reaction mixture, and the samples were incubated at room temperature in the dark for 30 min. Fluorescence from each sample was measured using an excitation wavelength of 530 nm and emission wavelength of 585 nm in a Biotek multimode plate reader. A six-point standard curve, ranging from 0 to 20 μM succinic acid, was prepared to calculate the amount of succinic in each sample. Sterile BSM was included as a positive control, whereas BSM without succinate was used as a negative control.

**Quantification of Nucleic Acid Synthesis**

The incorporation of [5,6-^3^H]-uridine (PerkinElmer/Revvity, Hopkinton, MA) into newly synthesized nucleic acids was measured in stationary phase *P. aeruginosa* and *E. coli.* 1 mL aliquots of each culture were prepared in 15 mL conical tubes containing 1 μCi of tritiated uridine. Thiolutin-treated samples were incubated with thiolutin at approximately the MIC for each species (100 μg/mL for *P. aeruginosa* or 8 μg/mL for *E. coli*) and tritiated uridine. Samples were incubated at 37 ^o^C, with shaking at 250 rpm, for 1 h.

After incubation, 2.7 mL of ice-cold 10% trichloroacetic acid (TCA) was added to each sample before incubating on ice for 30 min. The precipitates from each sample were collected by vacuum filtration onto 25 mm Whatman GF/C glass filters. These filters were pre-wetted with 10% TCA before being overlaid on a vacuum filter holder with a borosilicate glass base. Precipitates on the filters were rinsed three times with 2.7 mL of ice-cold 10% TCA and three times with 2.7 mL of ice-cold 70% ethanol. The filters were then air dried for 2 h before they were transferred into 6-mL scintillation vials containing 5 mL of EconoSafe scintillation fluid. After 10 min, radioactivity was measured using a Beckman-Coulter LS6500 liquid scintillation counter. Counts per minute (CPM) of each sample was subtracted with CPM from the negative control, which contained an unused Whatman GF/C glass filter in scintillation fluid. These background-subtracted CPM values were normalized to the OD_600_ of each sample.

As an additional negative control, 0.1 volume (110 μL) of 3 M KOH was added to replicate aliquots of cells that had been incubated with radioactive uridine. These samples were incubated statically at 37 ^o^C for 24 h to hydrolyze labile RNA. Following this incubation period, 2.7 mL of ice-cold TCA was added to these samples, and they were chilled on ice for 30 min. The precipitates were collected by vacuum filtration, washed, and scintillated following the same protocol as for other samples.

**Measuring Protein Translation Activity with Click-iT AHA**

Protein translation was measured as previously described (10). In brief, 500 μL aliquots of exponentially growing or stationary-phase *P. aeruginosa* or *E. coli* were transferred into 15 mL conical tubes with either i) no reagent, ii.) 1 mM L-Azidohomoalanine (AHA; Thermofisher C10102), iii) 1 mM methionine (100 mM stock), or iv) 1 mM AHA plus 1 mM methionine and incubated for 1 h at 37°C, shaking at 250 rpm. Then, samples were centrifuged for 3 min at 21,000 x g, resuspended in PBS, transferred into 7 mL 90% ice-cold methanol for fixation, and stored at -20 ^o^C until analysis.

To prepare the samples for click chemistry reactions, each tube was centrifuged at 4 ^o^C for 10 min at 3,220 x g and ~7 mL supernatant was removed. Cell pellets were resuspended in the remaining 500 μL supernatant and transferred to microcentrifuge tubes for additional centrifugation for 3 min at 21,000 x g. All supernatant was removed, pellets were washed in 500 μL PBS, and then centrifuged and resuspended in 100 μL 0.5% Triton-X in PBS for 30 min at room temperature. After 30 min, samples were washed once with 500 μL PBS then resuspended in 100 μL reaction mix consisting of 2.5 μM AlexaFluor 647 Alkyne (5 mM stock in DMSO for working concentration; ThermoFisher A10279) and 2 μM CuSO_4_ (100 mM stock) in 1X Click-iT buffer additive in PBS (ThermoFisher C10424). Reactions were performed in the dark at room temperature for 30 min. After 30 min, samples were centrifuged and resuspended in 500 μL PBS. Samples were diluted to OD_600_ ~0.01 in PBS for analysis by flow cytometry. Samples were analyzed on an LSRII flow cytometer with FACS DiVa (BD Biosciences) with a filter for red fluorescence (670/30 nm band-pass). Data were analyzed using FlowJo (BD Biosciences). To quantify the percent of fluorescent cells in each population, a fluorescence-negative gate was set to capture ~95% of the methionine-fed population. Cells fed AHA with fluorescence above this threshold were determined to be fluorescence-positive. Fluorescence intensity was quantified as the median signal of AHA-fed cells that were fluorescence-positive according to the prior gating.

**Table S1.** Minimum inhibitory concentrations (mean + standard error of the mean) as measured by the MIC test strip method (n>3).

| Strain | Levofloxacin (μg/mL) | Tobramycin (μg/mL) | Aztreonam (μg/mL) |
| --- | --- | --- | --- |
| *P. aeruginosa* PAO1 | 0.347 + 0.045 | 0.563 + 0.110 | 3.67 + 0.33 |
| *P. aeruginosa* PA14 | 0.230 + 0.020 | 0.667 + 0.083 | 4.33 + 0.88 |

**Table S2.** Bacterial strains used in this study.

| Strain (Genotype) | Description | Source |
| --- | --- | --- |
| *E. coli* MG1655 | F^-^ λ^-^ *ilvG*^-^ *rfb*-50 *rph*-1 | ATCC 700926 |
| *P. aeruginosa* PAO1 | wild-type | Poole Lab,  Queen’s University(11) |
| PAO1 Δ*recA* | *recA* knockout | This work |
| *P. aeruginosa* PA14 | wild-type  NR-50573 | BEI Resources |
| PA14 Δ*recA* | *recA* knockout | This work |
| *E. coli* HB101::pRK2013 | mobilization helper plasmid; Kan^R^ | Wu Orr Lab,  Amherst College |
| *E. coli* DH5α::pEX18Gm | Broad host range *sacB* suicide cloning vector for allelic exchange; Gent^R^ | Wu Orr Lab,  Amherst College |

**Table S3.** PCR Primers used for this study.

| Primer | Sequence (5' to 3') |
| --- | --- |
| *recA*_up_FWD | tcatcatACTAGTGAATTCTTCGGGCTGGTTCGAGGCTG |
| *recA*_up_REV | CTTCGGCGTCGGCCTTCACCCTTCTTGTTCTCGTCCAT |
| *recA*_down_FWD | GGTGAAGGCCGACGCCGAAG |
| *recA*_down_REV | gtagtag***GGATCC***AAGCTTAACTGGGACTGAAGCTGCCG |
| *recA*_ext_FWD2 | AGTGAGCGCTGCCAGTTC |
| *recA*_ext_REV | CGAACCGGAGTTCCGCAACC |
| *recA*_ext_REV2 | GGGCAGGATCGATCAACT |
| *recA*_int_FWD2 | CCACCTGTGCCTTCGTCG |
| *recA*_int_REV2 | CTTCTCCAGTACCGAACCGA |

The ~500 bp regions surrounding *recA* were amplified using primer pairs *recA*_up_FWD / *recA*_up_REV and *recA*_down_FWD / *recA*_down_REV using PAO1 or PA14 genomic DNA as a template. The upstream and downstream regions were joined in overlap PCR using *recA*_up_FWD and *recA*_down_REV. The PCR product and the pEX18Gm vector were both digested with EcoRI and BamHI (restriction sites are underlined and bolded/italicized, respectively). The PCR product insert was then ligated into the opened pEX18Gm backbone to create the vectors pEX18Gm-PAO1*-recA*KO or pEX18Gm-PA14-*recA*KO.

Putative PAO1 *recA* knockout clones were screened by colony PCR using the primer pairs *recA*_int_FWD2 / *recA*_int_REV2 and *recA*_ext_FWD2 / *recA*_ext_REV2. Putative PA14 *recA* knockouts were screened using *recA*_int_FWD2 / *recA*_int_REV2 and *recA*_ext_FWD2 / *recA*_ext_REV.

**Table S4.** Minimum inhibitory concentrations as measured by broth microdilution assay in CA-MHB (n=2).

| Strain | Thiolutin (μg/mL) | Thiourea (mM) | 2,2'-bipyridine (mM) |
| --- | --- | --- | --- |
| *P. aeruginosa* PAO1 | 128 | 150 | 5 |
| *P. aeruginosa* PA14 | 128 | 150 | 2.5 – 5 |
| *E. coli* MG1655 | 8 | - | - |

**
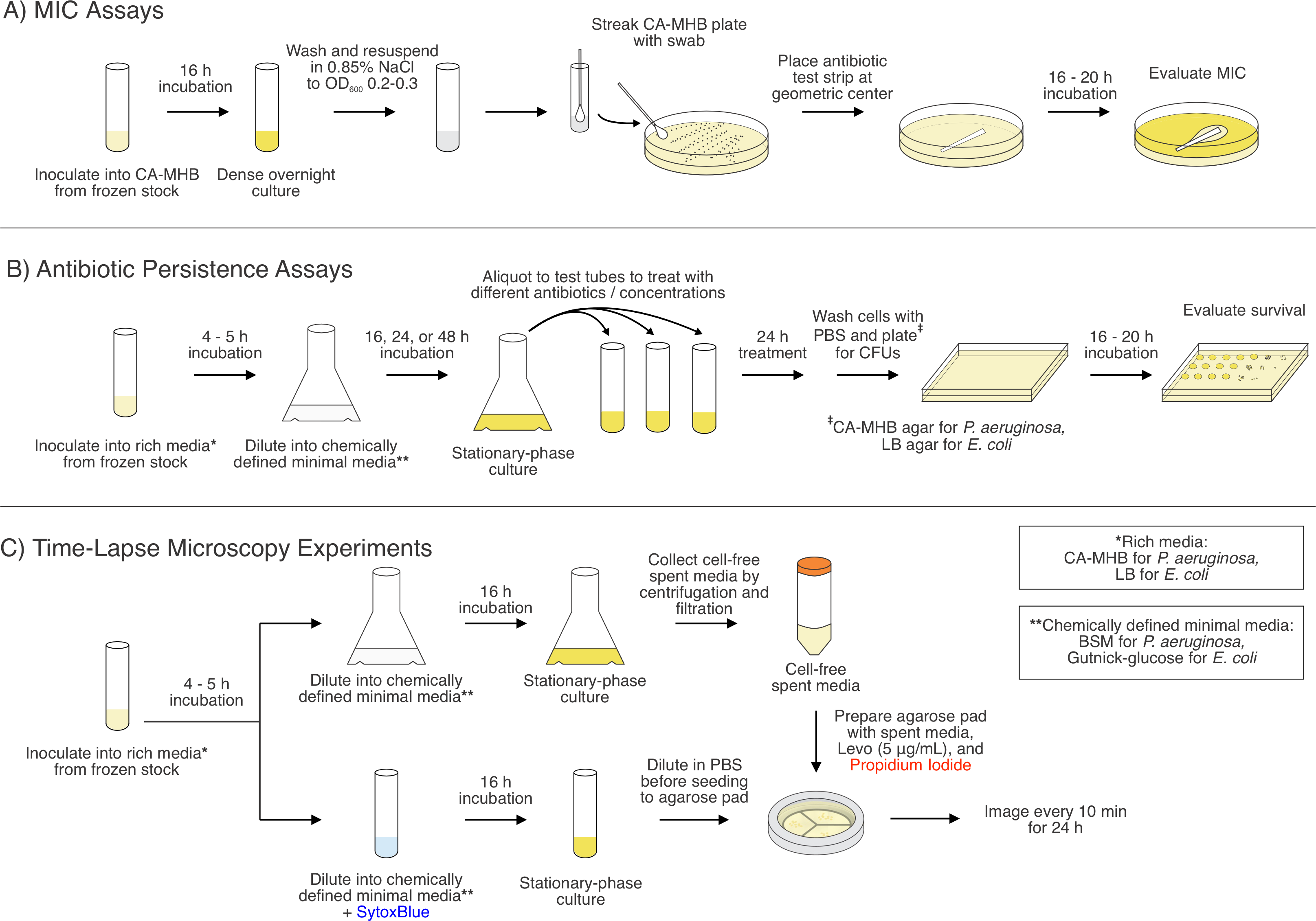
**

**Figure S1.** Diagrams depict the core experimental designs and workflows in this manuscript. MIC: minimum inhibitory concentration; CA-MHB: cation-adjusted Mueller-Hinton broth; BSM: Basal Salt Media; LB: lysogeny broth.

**A**) Minimum inhibitory concentration (MIC) tests for *P. aeruginosa* with the antibiotics Levo, Tobramycin, and Aztreonam were conducted using the MIC test strip method.

**B**) For antibiotic persistence assays, cells were grown to stationary phase in chemically defined media before treatment with antibiotics. Survival was calculated based on the colony-forming units per milliliter (CFU/mL) of each sample after 24 h treatment compared to the culture’s pre-treatment CFU/mL.

**C**) Preparation for time-lapse microscopy of cells during Levo treatment involved two parallel cultures: one culture for cell-free spent media collection to prepare the agarose pad (top) and another culture for cells to be stained with SytoxBlue and imaged (bottom). The SytoxBlue-to-propidium iodide (PI) switch allowed us to determine if cell death occurred during the growth to stationary phase (SytoxBlue-positive) or whether it occurred after exposure to Levo in the agarose pad (PI-positive).


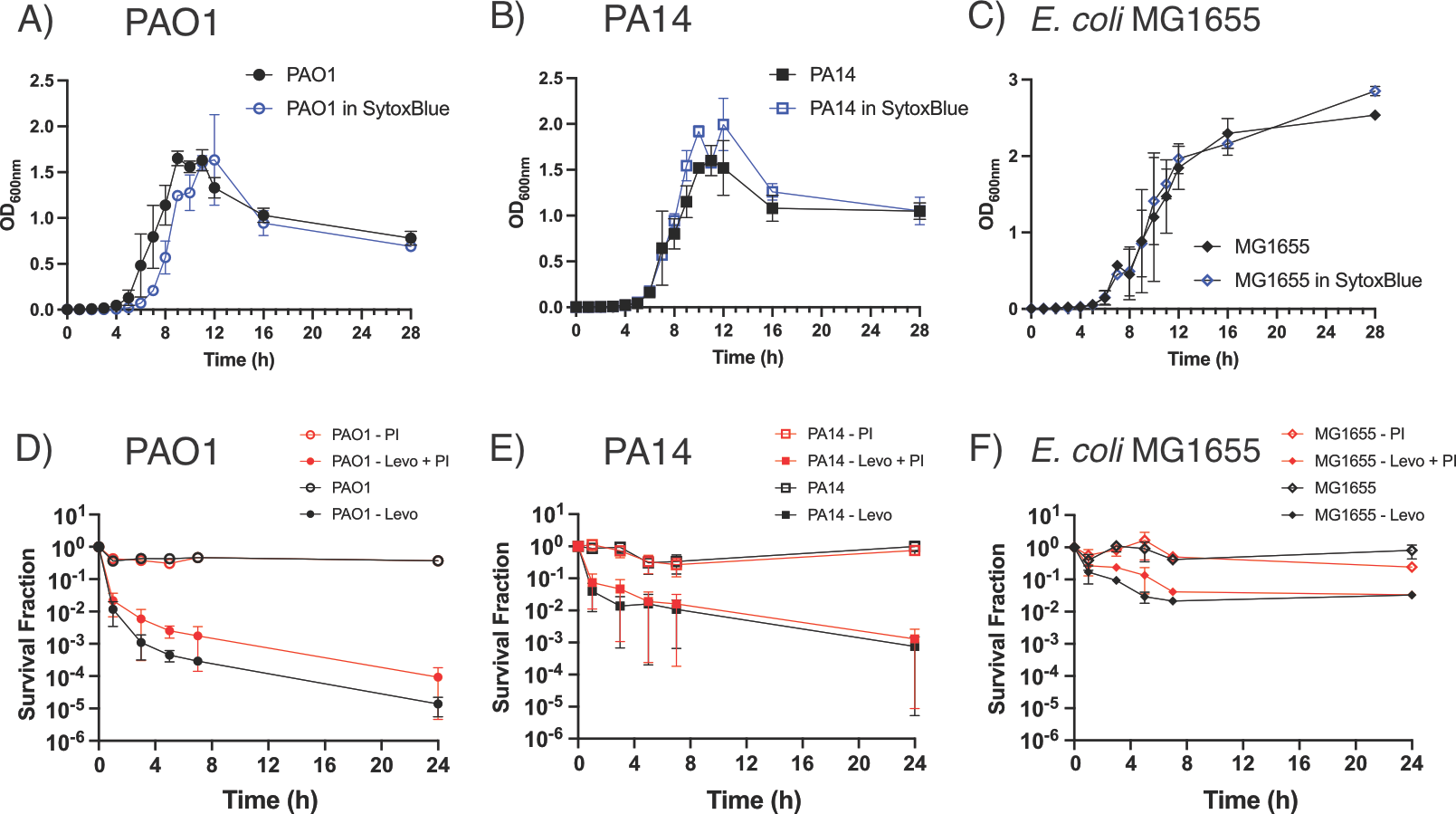


**Figure S2.** The SytoxBlue and propidium iodide concentrations used for time-lapse microscopy experiments do not affect cell viability during growth or treatment.

**Top panel:** The semi-permeable dye, SytoxBlue (2.5 μM), does not alter the growth of **A**) *P. aeruginosa* PAO1, **B**) PA14, or **C**) *E. coli* MG1655 to stationary phase in their respective chemically defined minimal media (BSM for *P. aeruginosa* and Gutnick Media [with 10 mM glucose] for *E. coli*). Data are representative of the mean and standard error of the mean of at least two biological replicates.

**Bottom panel:** Propidium iodide (16 μM) does not alter the viability of stationary-phase **D**) *P. aeruginosa* PAO1, **E**) PA14, or **F**) *E. coli* MG1655 when cultures are either untreated (hollow symbols) or treated with 5 μg/mL Levo (solid symbols). These are the conditions used for time-lapse microscopy experiments and demonstrate that these dyes do not affect the viability of cells. Data are representative of the mean and standard error of the mean for three biological replicates.

**
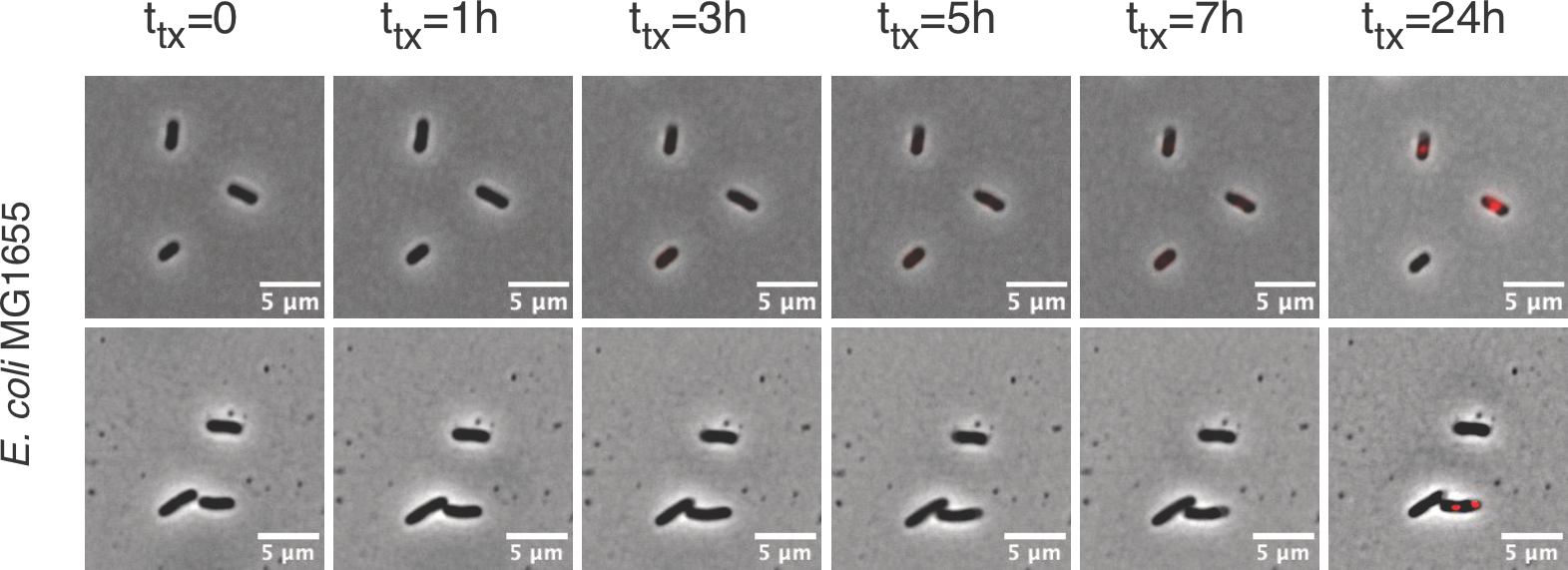
**

**Figure S3.** Stationary-phase *E. coli* cell morphologies are unchanged throughout Levo treatment.

Representative images of *E. coli* MG1655 during Levo treatment (5 μg/mL). The images depict the phase channel merged with the channel for propidium iodide (shown in red). Images are representative of two biological replicates.

**
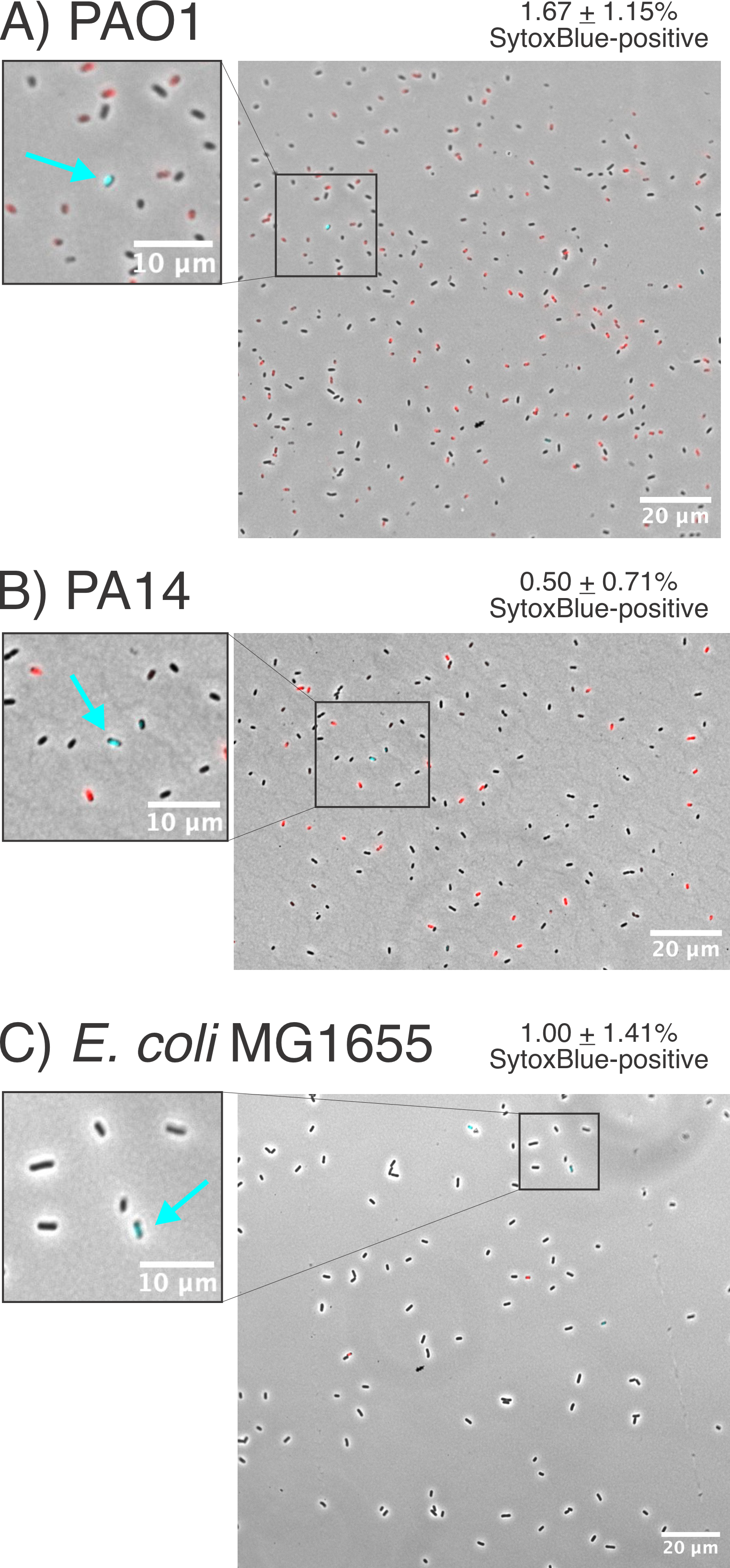
**

**Figure S4.** The majority of stationary-phase *P. aeruginosa* and *E. coli* from 16 h cultures are alive before Levo treatment.

Representative images of wild-type **A)** *P. aeruginosa* PAO1 (n=3 biological replicates), **B)** *P. aeruginosa* PA14 (n=2), and **C)** *E. coli* MG1655 (n=2) at the start of time-lapse imaging. The images depict the phase channel merged with channels for propidium iodide and SytoxBlue (shown in red and cyan, respectively). The percentage of SytoxBlue-positive cells is shown as mean + standard deviation.

**
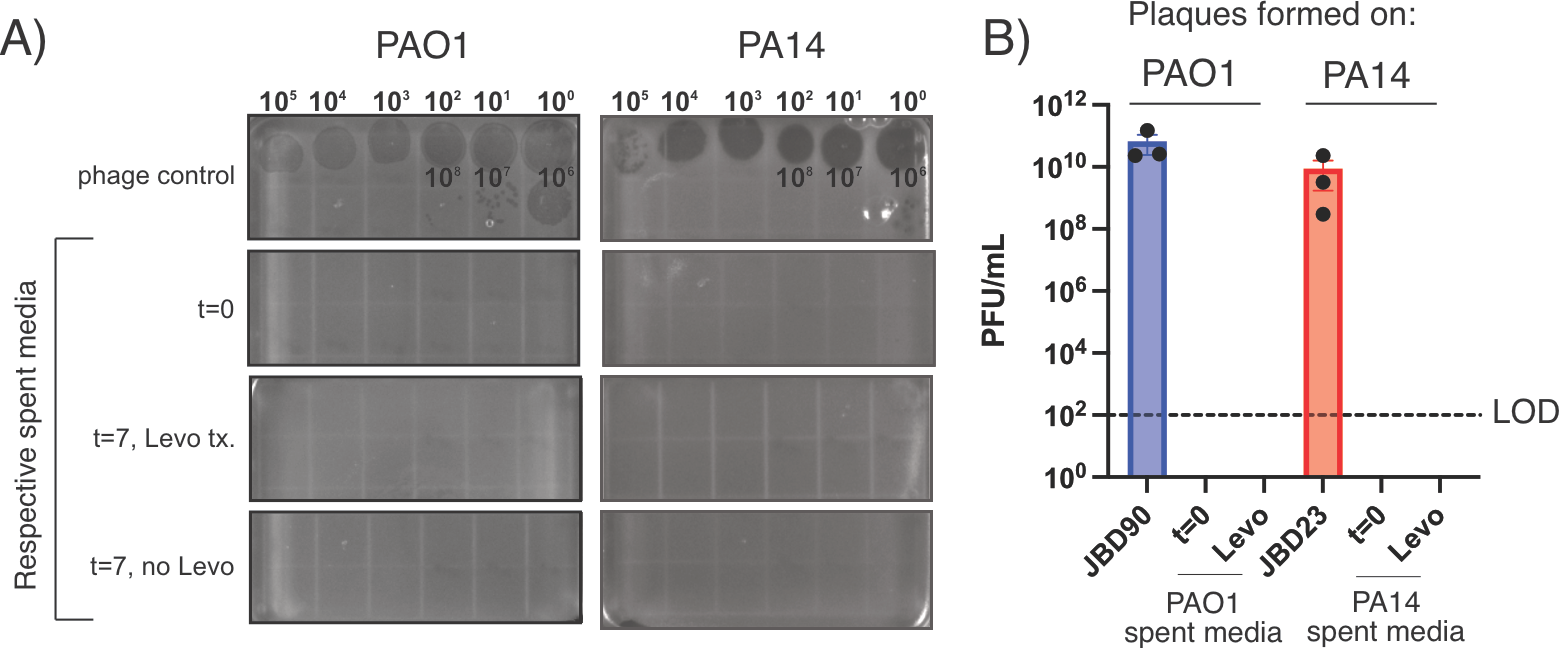
**

**Figure S5.** The conditioned media from Levo-treated *P. aeruginosa* is not sufficient to cause phage plaque formation.

**A)** Images depict phage plaque assay plates after overnight incubation. Plaque assays were conducted using the cell-free spent media from *P. aeruginosa* cultures before treatment or after 7 h Levo treatment. As controls, phage specific to PAO1 (JBD90) and PA14 (JBD23) were included. Images are representative of three biological replicates.

**B)** Quantification of plaque forming units per milliliter (PFU/mL) showed high titers for the control phages but no detectable PFU/mL for the spent media from PAO1 or PA14 before treatment or after 7 h Levo treatment, suggesting that the observed cell lysis during Levo treatment is not due to induction of prophages. The limit of detection (LOD) is denoted by the dashed line.


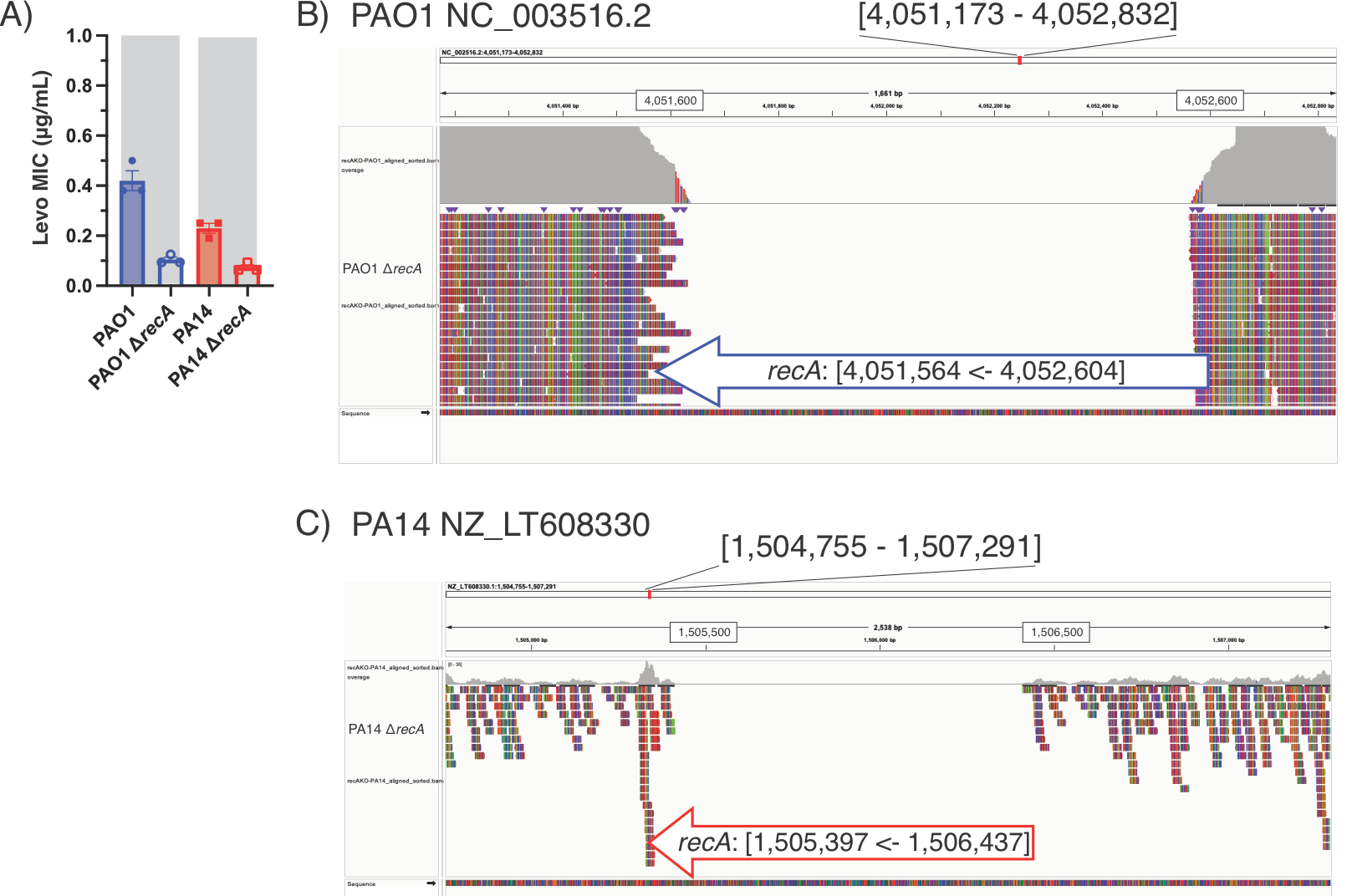


**Figure S6.** *P. aeruginosa* Δ*recA* strains are validated by phenotypic screening by MIC assay and whole-genome sequencing.

**A)** The minimum inhibitory concentration of Levo, measured by the MIC test strip method, is lower for *recA* deletion strains than the wild-type parental strains (n=3). This is concordant with previous literature (12, 13). The gray boxes denote the CLSI MIC susceptibility breakpoint for Levo for *P. aeruginosa* (1 µg/mL) (14).

**B** and **C)** Integrative Genomics Viewer (IGV) browser sessions demonstrating that no reads from whole genome sequencing of PAO1 Δ*recA* or PA14 Δ*recA* map to *recA* in the respective reference genomes (PAO1: NCBI NC_002516.2; PA14: NCBI NZ_LT608330) (15).

**
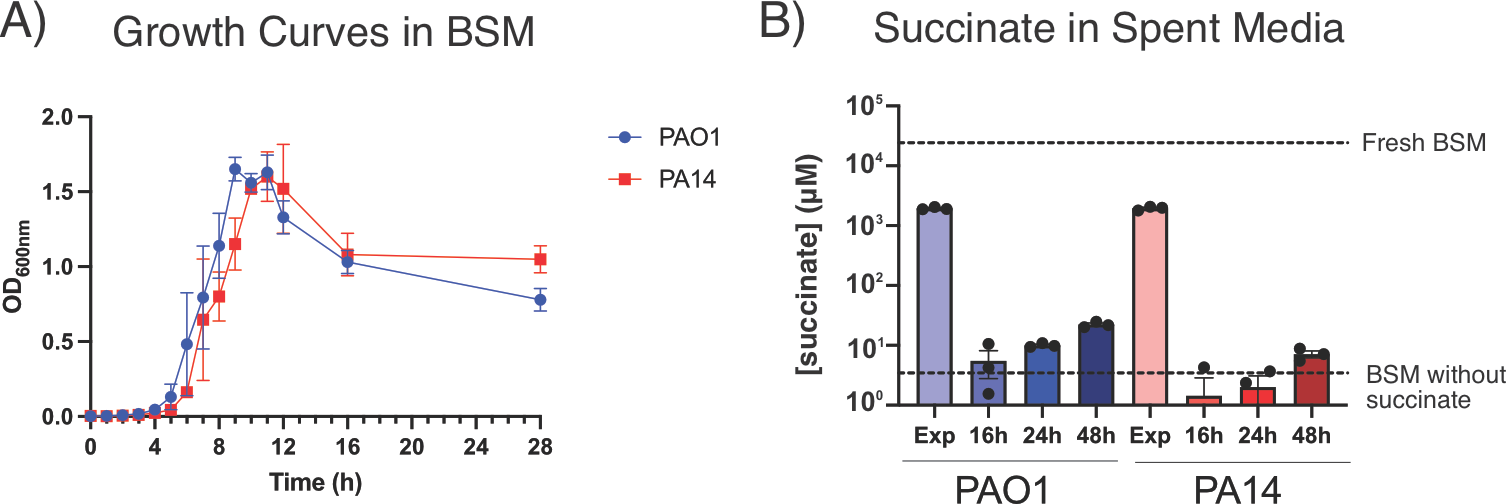
**

**Figure S7.** The culture conditions used in this study are sufficient to cultivate *P. aeruginosa* to stationary phase.

**A)** Measurement of OD_600_ over time demonstrates that *P. aeruginosa* PAO1 and PA14 cultures in Basal Salt Media (BSM) have stopped growing by 16 h, which we define as our standard stationary-phase culture conditions. These cultures were inoculated at a starting OD just above the spectrophotometer limit of detection (between 0.001-0.005) and even still, the terminal OD is reached before 16 h. Data represent the mean from three biological replicates and standard error of the mean.

**B)** At the designated times, cultures were filtered and the succinic acid concentrations were measured by fluorometric assay. The upper limit on the graph depicts 15 mM succinic acid (the concentration in fresh BSM) and the lower limit represents BSM salts without succinic acid. By 16 h, the succinic acid levels approximate that of BSM without any succinic acid for both PAO1 and PA14. Data shown represent three biological replicates with standard error of the mean.

**
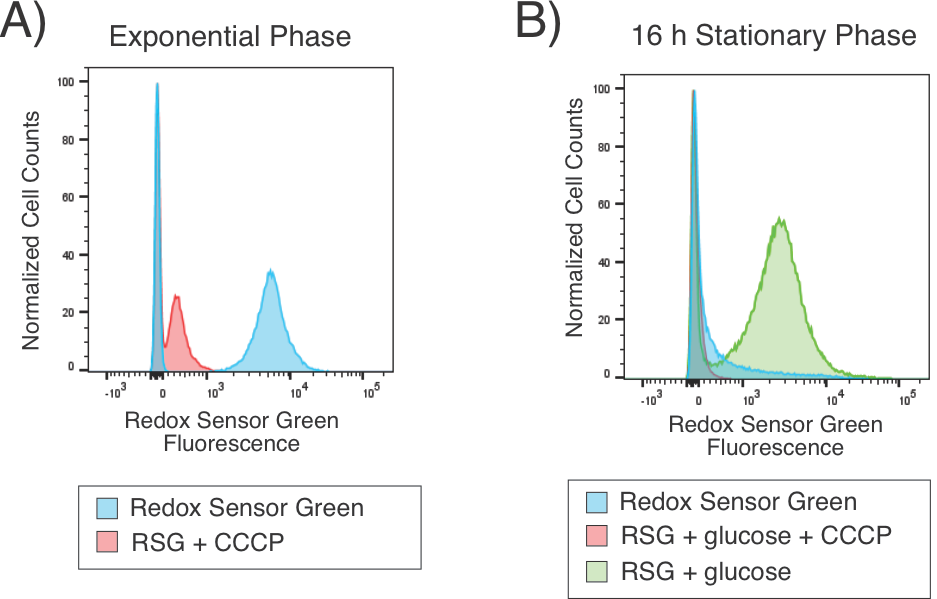
**

**Figure S8.** *E. coli* MG1655 has reductase activity in exponential-phase, but not stationary-phase, cultures.

**A)** Exponential-phase *E. coli* MG1655 has reductase activity, as measured by flow cytometry after dying with Redox Sensor Green (RSG). The addition of CCCP (2.5 μM) abrogates the signal.

**B)** Stationary-phase (16 h) cultures of MG1655 have very diminished reducing activity unless metabolically stimulated with 10 mM glucose. Three replicates were performed for these experiments.

**
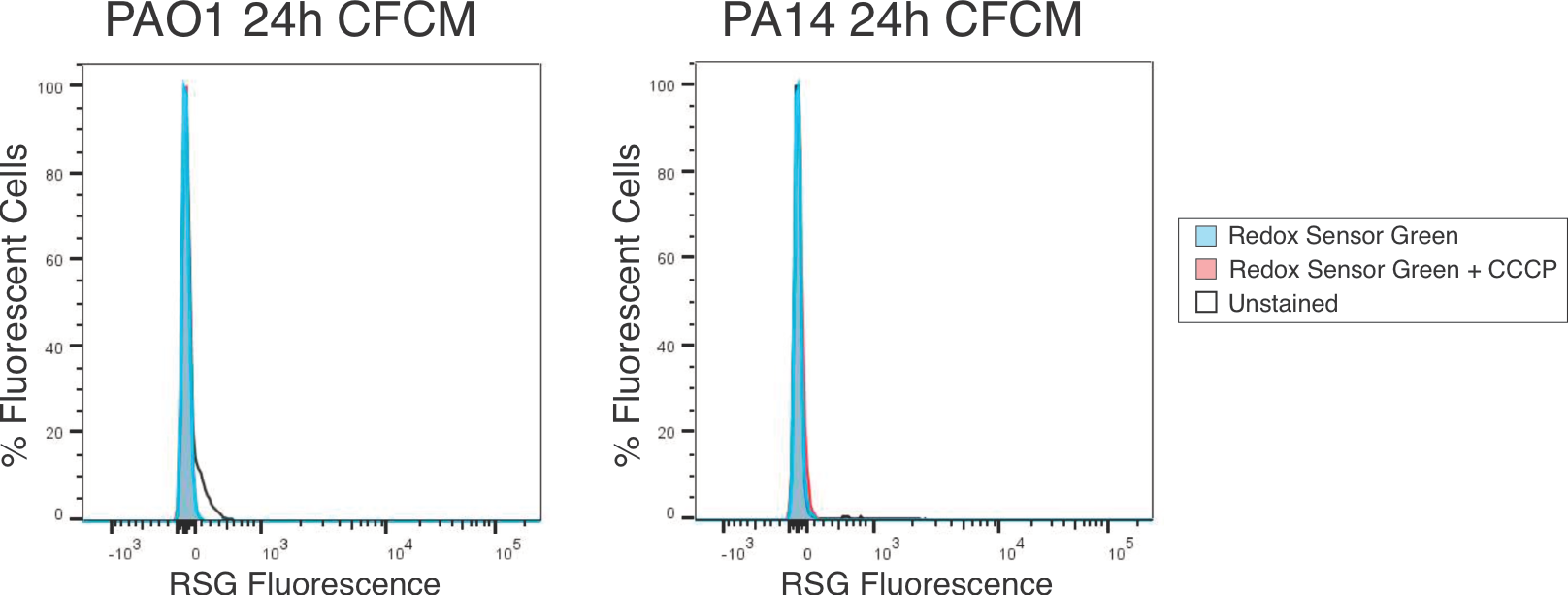
**

**Figure S9.** The secreted products from *P. aeruginosa* cultures is insufficient to cause Redox Sensor Green (RSG) fluorescence.

Cell-free conditioned media (CFCM) is free of redox-active metabolites. CFCM from 24 h cultures of PAO1 and PA14 were stained for RSG with or without CCCP at the same concentrations/ratios used for the cell samples shown in Figure 4. Unstained CFCM was included as a negative control. The lack of signal from the CFCM suggests that RSG signal observed for PAO1 and PA14 cell cultures is due to reductase activity in the cells and not extracellular redox cycling molecules. Histograms are representative of two biological replicates.

**
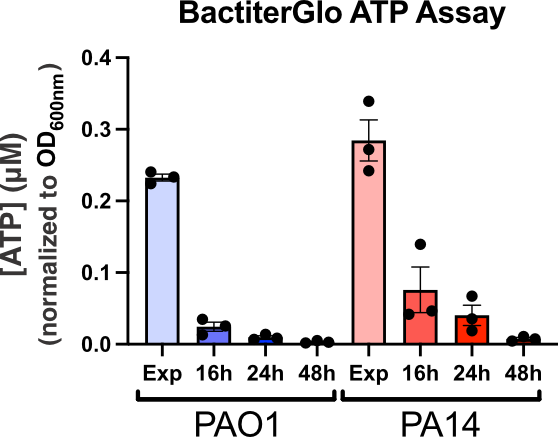
**

**Figure S10.** Stationary-phase *P. aeruginosa* cells are deenergized.

ATP levels were quantified for cultures of PAO1 or PA14 at the designated times with the BactiterGlo luminescence assay. Data represent three biological replicates.


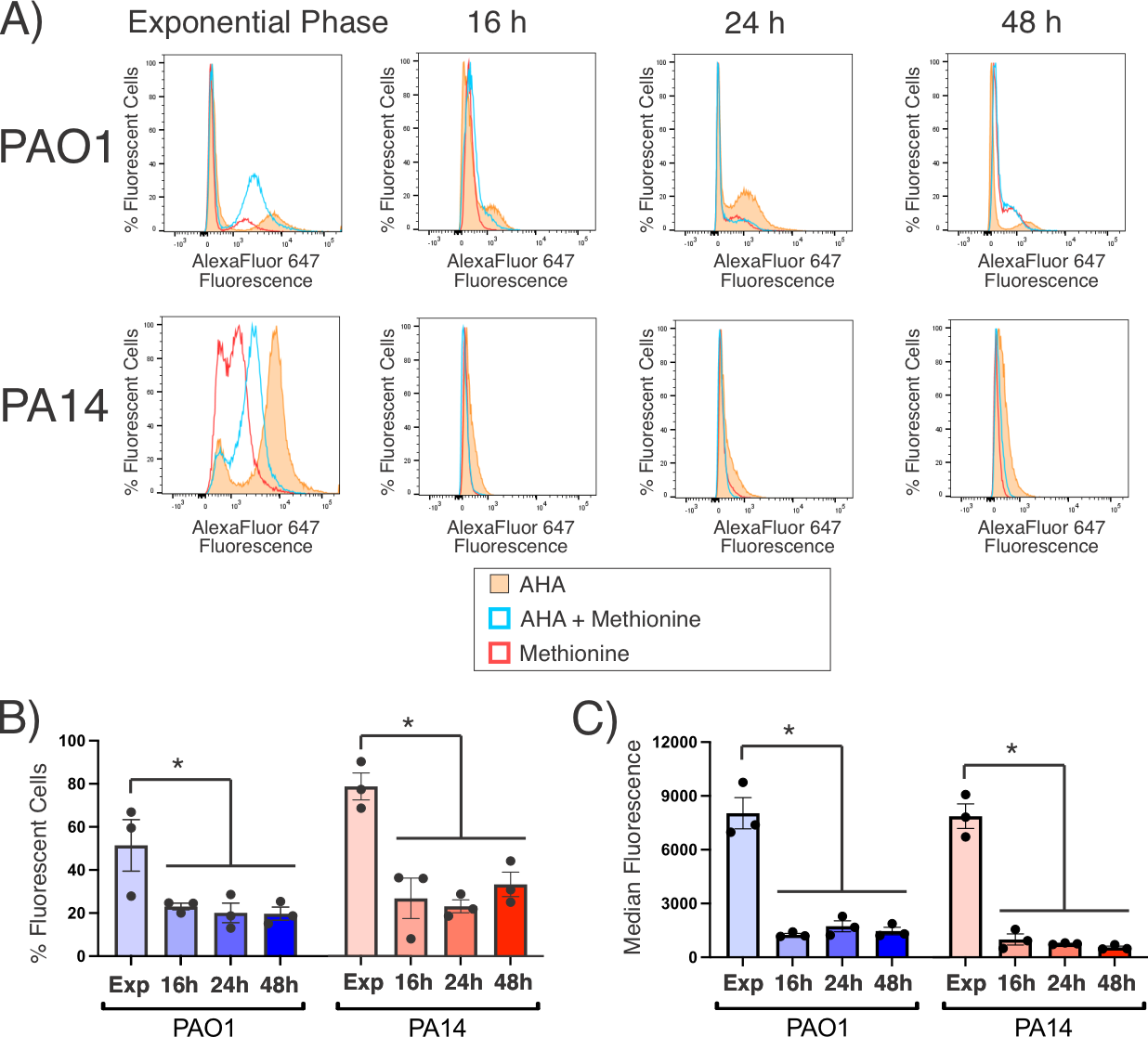


**Figure S11.** Stationary-phase *P. aeruginosa* cultures have reduced translational activity.

**A)** Protein translation activities were assessed in *P. aeruginosa* cultures grown to exponential phase or stationary phase (16, 24, or 48 h) by measuring the incorporation of an azidated methionine analog, azidohomoalanine (AHA), that was fluorescently labeled with alkyne AlexaFluor 647. Addition of unlabeled methionine or a 1:1 mixture of methionine:AHA served as negative controls. Histograms are representative of three biological replicates.

**B)** The percentage of fluorescent cells was quantified for each strain and time point. The asterisks designate that exponential-phase cultures have significantly more fluorescent cells than stationary-phase cultures (one-way ANOVA, p < 0.05).

**C)** The signal intensity of fluorescent cells are significantly higher for exponential-phase cultures compared to the stationary-phase cultures for both strains, as indicated by the asterisks (one-way ANOVA, p < 0.05). Together, these data suggest that stationary-phase cultures have both fewer translating cells and lower levels of translation compared to exponential-phase cultures.

**SUPPLEMENTAL VIDEO CAPTIONS**

**Video S1. PAO1_WT_5ug-mL-Levo_1.avi**

24 h time-lapse video of wild-type *P. aeruginosa* PAO1 during Levo treatment. PAO1 was grown for 16 h to stationary phase in BSM with SytoxBlue (2.5 µM) in test tubes before seeding onto an agarose pad (1.5%) made from its own 16 h cell-free conditioned media plus Levo (5 µg/mL) and propidium iodide (16 µM). Images were taken for the phase and OFP channels (for propidium iodide) every 10 min for 24 h. Images were acquired with a pco.panda 4.2 bi sCMOS camera (6.5 μm pixel size).

**Video S2. PAO1_WT_5ug-mL-Levo_2.avi**

24 h time-lapse video of wild-type *P. aeruginosa* PAO1 during Levo treatment (second biological replicate). Same methods as for replicate 1 (**Video S1**).

**Video S3. PA14_WT_5ug-mL-Levo_1.avi**

24 h time-lapse video of wild-type *P. aeruginosa* PA14 during Levo treatment. PA14 was grown for 16 h to stationary phase in BSM with SytoxBlue (2.5 µM) in test tubes before seeding onto an agarose pad (1.5%) made from its own 16 h cell-free conditioned media plus Levo (5 µg/mL) and propidium iodide (16 µM). Images were taken for the phase and OFP channels (for propidium iodide) every 10 min for 24 h. Images were acquired with a Hamamatsu ORCA-R2 camera (6.45 μm pixel size).

**Video S4. PA14_WT_5ug-mL-Levo_2.avi**

24 h time-lapse video of wild-type *P. aeruginosa* PA14 during Levo treatment (second biological replicate). Same methods as for replicate 1 (**Video S3**).

**Video S5. Ecoli_MG1655_5ug-mL-Levo_1_2.mov**

24 h time-lapse videos of wild-type *E. coli* MG1655 during Levo treatment (replicates 1 and 2). *E. coli* MG1655 was grown for 16 h to stationary phase in Gutnick-glucose media with SytoxBlue (2.5 µM) in test tubes before seeding onto an agarose pad (1.5%) made from its own 16 h cell-free conditioned media plus Levo (5 µg/mL) and propidium iodide (16 µM). Images were taken for the phase and OFP channels (for propidium iodide) every 10 min for 24 h. Images for replicate 1 were acquired with a pco.panda 4.2 bi sCMOS camera (6.5 μm pixel size). Images for replicate 2 were acquired with a Hamamatsu ORCA-R2 camera (6.45 μm pixel size).

**Video S6. PAO1_recAKO_5ug-mL-Levo_1.avi**

24 h time-lapse video of *P. aeruginosa* PAO1 Δ*recA* during Levo treatment. PAO1 Δ*recA* was grown for 16 h to stationary phase in BSM in test tubes before seeding onto an agarose pad (1.5%) made from its own 16 h cell-free conditioned media plus Levo (5 µg/mL) and propidium iodide (16 µM). Images were taken for the phase and OFP channels (for propidium iodide) every 10 min for 24 h. Images were acquired with a pco.panda 4.2 bi sCMOS camera (6.5 μm pixel size).

**Video S7. PAO1_recAKO_5ug-mL-Levo_2.avi**

24 h time-lapse video of *P. aeruginosa* PAO1 Δ*recA* during Levo treatment (second biological replicate). Same methods as for the first replicate (**Video S7**).

**Video S8. PA14_recAKO_5ug-mL-Levo_1.avi**

24 h time-lapse video of *P. aeruginosa* PA14 Δ*recA* during Levo treatment. PA14 Δ*recA* was grown for 16 h to stationary phase in BSM in test tubes before seeding onto an agarose pad (1.5%) made from its own 16 h cell-free conditioned media plus Levo (5 µg/mL) and propidium iodide (16 µM). Images were taken for the phase and OFP channels (for propidium iodide) every 10 min for 24 h. Images were acquired with a Hamamatsu ORCA-R2 camera (6.45 μm pixel size).

**Video S9. PA14_recAKO_5ug-mL-Levo_2.avi**

24 h time-lapse video of *P. aeruginosa* PA14 Δ*recA* during Levo treatment (second biological replicate). Same methods as for the first replicate (**Video S9**).

**REFERENCES**

1. Robinson JL, Jaslove JM, Murawski AM, Fazen CH, Brynildsen MP. 2017. An integrated network analysis reveals that nitric oxide reductase prevents metabolic cycling of nitric oxide by *Pseudomonas aeruginosa*. Metab Eng 41:67–81.

2. Wolff JA, MacGregor CH, Eisenberg RC, Phibbs P V. 1991. Isolation and characterization of catabolite repression control mutants of *Pseudomonas aeruginosa* PAO. J Bacteriol 173:4700-4706.

3. Byrd BA, Zenick B, Rocha-Granados MC, Englander HE, Hare PJ, LaGree TJ, DeMarco AM, Mok WWK. 2021. The AcrAB-TolC efflux pump impacts persistence and resistance development in stationary-phase *Escherichia coli* following delafloxacin treatment. Antimicrob Agents Chemother 65:e00281-21.

4. Gutnick D, Calvo JM, Klopotowski T, Ames BN. 1969. Compounds which serve as the sole source of carbon or nitrogen for *Salmonella typhimurium* LT-2. J Bacteriol 100:215-219.

5. Hmelo LR, Borlee BR, Almblad H, Love ME, Randall TE, Tseng BS, Lin C, Irie Y, Storek KM, Yang JJ, Siehnel RJ, Howell PL, Singh PK, Tolker-Nielsen T, Parsek MR, Schweizer HP, Harrison JJ. 2015. Precision-engineering the *Pseudomonas aeruginosa* genome with two-step allelic exchange. Nat Protoc 10:1820–1841.

6. Peng T, Thorn K, Schroeder T, Wang L, Theis FJ, Marr C, Navab N. 2017. A BaSiC tool for background and shading correction of optical microscopy images. Nat Commun 8:14836.

7. Parslow A, Cardona A, Bryson-Richardson RJ. 2014. Sample drift correction following 4D confocal time-lapse imaging. J Vis Exp 86:e51086.

8. Ducret A, Quardokus EM, Brun Y V. 2016. MicrobeJ, a tool for high throughput bacterial cell detection and quantitative analysis. Nat Microbiol 1:16077.

9. Bondy-Denomy J, Qian J, Westra ER, Buckling A, Guttman DS, Davidson AR, Maxwell KL. 2016. Prophages mediate defense against phage infection through diverse mechanisms. ISME J 10:2854.

10. LaGree TJ, Byrd BA, Quelle RM, Schofield SL, Mok WWK. 2023. Stimulating transcription in antibiotic-tolerant *Escherichia coli* sensitizes it to fluoroquinolone and nonfluoroquinolone topoisomerase inhibitors. Antimicrob Agents Chemother 67:e0163922.

11. Masuda N, Ohya S. 1992. Cross-resistance to meropenem, cephems, and quinolones in *Pseudomonas aeruginosa*. Antimicrob Agents Chemother 36:1847-1851.

12. Mercolino J, Sciuto A Lo, Spinnato MC, Rampioni G, Imperi F. 2022. RecA and specialized error-prone DNA polymerases are not required for mutagenesis and antibiotic resistance induced by fluoroquinolones in *Pseudomonas aeruginosa*. Antibiotics 11:325.

13. Valencia EY, Esposito F, Spira B, Blázquez J, Galhardo RS. 2017. Ciprofloxacin-mediated mutagenesis is suppressed by subinhibitory concentrations of amikacin in *Pseudomonas aeruginosa*. Antimicrob Agents Chemother 61:e02107-16.

14. Clinical and Laboratory Standards Institute. 2021. Performance standards for antimicrobial susceptibility testing. 31st ed, Wayne, PA.

15. Robinson JT, Thorvaldsdóttir H, Winckler W, Guttman M, Lander ES, Getz G, Mesirov JP. 2011. Integrative genomics viewer. Nat Biotechnol 29:24-26.
